# Evaluation and comparison of classical interatomic potentials through a user-friendly interactive web-interface

**DOI:** 10.1038/sdata.2016.125

**Published:** 2017-01-31

**Authors:** Kamal Choudhary, Faical Yannick P. Congo, Tao Liang, Chandler Becker, Richard G. Hennig, Francesca Tavazza

**Affiliations:** 1 Materials Science and Engineering Division, National Institute of Standards and Technology, Gaithersburg, Maryland 20899, USA; 2 Department of Materials Science and Engineering, The Pennsylvania State University, University Park, pennsylvania 16801, USA; 3 Office of Data and Informatics, National Institute of Standards and Technology, Gaithersburg, Maryland 20899, USA; 4 Department of Materials Science and Engineering, University of Florida, Gainesville, Florida 32611, USA

**Keywords:** Condensed-matter physics, Computational methods, Mechanical properties

## Abstract

Classical empirical potentials/force-fields (FF) provide atomistic insights into material phenomena through molecular dynamics and Monte Carlo simulations. Despite their wide applicability, a systematic evaluation of materials properties using such potentials and, especially, an easy-to-use user-interface for their comparison is still lacking. To address this deficiency, we computed energetics and elastic properties of variety of materials such as metals and ceramics using a wide range of empirical potentials and compared them to density functional theory (DFT) as well as to experimental data, where available. The database currently consists of 3248 entries including energetics and elastic property calculations, and it is still increasing. We also include computational tools for convex-hull plots for DFT and FF calculations. The data covers 1471 materials and 116 force-fields. In addition, both the complete database and the software coding used in the process have been released for public use online (presently at http://www.ctcms.nist.gov/∼knc6/periodic.html) in a user-friendly way designed to enable further material design and discovery.

## Background & Summary

Classical^1^ or quantum mechanical^2^ atomistic simulations provide important insights into material phenomena, and, therefore, are a fundamental tool for material design. While quantum mechanical calculations are capable of providing a much more accurate description of materials, their applications are limited because their high computational cost limits both the size and the time length of the simulations.

Classical empirical potentials can be used to model much larger systems (millions of atoms), with varying temperature and for longer time, enabling, for instance, the investigation of point, line and planar defects. However, one of the biggest drawbacks of classical simulations is that results are only as good as the force-field used to obtain them. Because of that, the two main problems related to the use of classical-mechanics simulations are the transferability and the version-control of the originally developed potentials.

Transferability is the ability of a potential to produce reliable results when simulating conditions different from those used in the fitting procedure. As it is challenging to generate perfectly transferable potentials, before starting an investigation it is crucial to determine whether a potential exists that is suitable for modelling the particular physical properties and system of interest^3^. Generally interatomic potentials are fit to a specific application and material. Bulk energetics, defects^4^, and mechanical properties^5–13^ are the most commonly fitted properties, especially for bulk solid-state materials. These quantities are obtained from experiments, when available, or from quantum mechanical calculations such as density functional theory (DFT) simulations. As only specific crystal structures are included in the fitting process, it is critically important to evaluate how well the same potential performs for other important crystal structures. Similarly, if only room temperature properties have been included in the fit, testing is necessary to evaluate the reliability at higher temperatures, and so on.

The second major concern related to the use of classical potentials is the possibility that the potential form actually implemented in the used code is not exactly identical to the form initially distributed by the developers. For instance, keeping the parameters intact over time is a challenging task due to fact that file formats change between codes and mistakes can be made in the transferring or the files themselves can get corrupted. Again, testing and benchmarking to the data reported in literature is therefore a needed practice before running new simulations.

In this work, we focus on developing a database and web interface to facilitate such transferability and reproducibility tests for classical empirical potentials. As a first step, we computed and compared energetics, convex hulls [26] and elastic constants (using the Large-scale Atomic/Molecular Massively Parallel Simulator( LAMMPS) provided script^14^) at T=0 K for all the following potentials: embedded atom method (EAM)^15^, embedded ion method (EIM)^16^, modified embedded atom methods (MEAM)^17^, AIREBO^18,19^, REAXFF^20^, charge-optimized many body (COMB)^21^, Tersoff^22^, Stillinger-Weber (SW) potentials^23,24^. Please note commercial software is identified to specify procedures. Such identification does not imply recommendation by the National Institute of Standards and Technology. Although, all the calculations done here at 0 K temperature, due to flexible framework of the high throughput calculation, it is easy to calculate temperature dependent properties. Temperature dependent properties calculations are subject to our future work. In addition to cross comparison among classical potentials, a direct comparison to DFT data obtained from the Materials-Project database^25^ is also provided. Such a database has been developed by the Persson group^25^ and provides DFT computation of structural, energetic, and elastic properties for thousands of materials. To facilitate the extraction of key information, we provide both our data and their DFT counterpart in the same easy look-up table on the web. We also make our generalized calculation set-up for the empirical potential data freely available on the web (https://github.com/JARVIS-Unifies/MPInterfaces). Users interested in benchmarking the available potentials can easily modify and tailor such a calculation set-up to their specific applications. We illustrate the usefulness of the dataset for users and developers of potentials by performing a principal-component analysis (PCA). The key feature of PCA is to reduce the dimensionality of data set consisting of large number of interrelated variables, while retaining as much as possible the variation present in dataset. PCA has been applied for various material problems such as 2-point spatial correlations of microstructure, rigorous structure quantification for molecular dynamics, phase field simulations of pattern formation and so on^26,27^. We use PCA on the relative errors of the predicted elastic constants compared to the experimental data. We find that the errors in the elastic constants from this script are correlated such that the error in *c*_44_ is about 2.5 times the sum of the errors in *c*_11_ and *c*_12_.

A significant amount of work towards assembling atomistic potential repositories and testing potentials is already present in the literature and online. Becker *et al.*^3^ developed the Interatomic potential repository (IPR) available on the National Institute of Standards and Technology (NIST) website, where examples of dissemination and evaluation of potentials are discussed, as well as tested potential parameters and files are available for download. OpenKIM^28^ is another important initiative dealing with assembly and testing of empirical potentials using application programme interface (API). However, these databases focus on potentials for metallic systems (mostly EAM) only. Therefore, the distribution of advanced potentials and of potentials for non-metallic systems, such as charge-optimize many body (COMB)^21^, Reaction force-field (REAXFF)^20^ and so on, is still lacking and it is one key component of this work. Also, there are very few evaluation of force-fields in previous works (e.g., 29). We provide the input and output files for all the calculations as well, for the LAMMPS software, to further facilitate the user in running similar benchmarking tests.

The paper is organized as follows: first the methods for developing this project are discussed, followed by data records and the important key elements of the data. After that, technical validation of the data is discussed using examples of single element, binary and ternary systems. Finally, usage notes have been discussed.

## Methods

**Figure 1:**
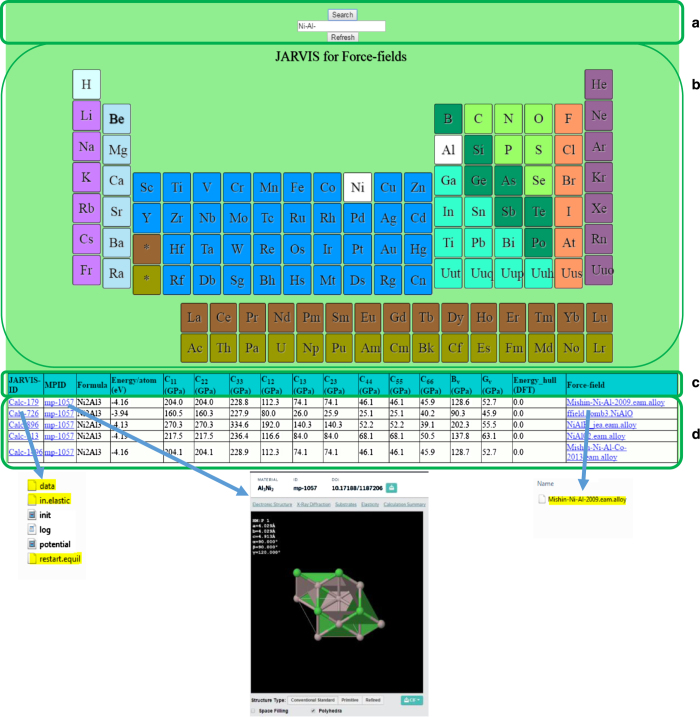
Interactive periodic table for searching potentials and corresponding calculations. List of options Ni-Al is shown as an example. Panel (**a**) shows the search box, panel (**b**) shows the interactive periodic table, panel (**c**) shows the header of the display table and the last panel (**d**) contains the actual data for all the quantities discussed in the header section.

The methodology behind the current work consisted of several steps:

1. **Obtaining structural data:** We started by downloading all the available potentials from the NIST interatomic potential repository (IPR) and from LAMMPS itself (15May2015 version). At present, there are 92 EAM, 9 Tersoff, 6 COMB, 5 ReaxFF, 2 AIREBO, 1 MEAM and 1 EIM potentials in the database. For each element having at least a potential, we downloaded all the corresponding crystal structures from the Materials Project (MP) database^30^. We also downloaded all the energetics and mechanical properties data from MP and stored them in a separate database for a later comparison with the classical results (LAMMPS calculations at T=0 K). More specifically, the download of the structures from MP was done using the Restful Application Program Interface (MP REST-API)^25,31^, while the python library URLLIB was used for downloading the potentials from IPR. A ‘from_ctcms.py’ script was used in this process and it is distributed in the github page of this project.
2. **Setting High-throughput framework:** The high-throughput setting of LAMMPS jobs was done using the MPinterfaces code^32^. Originally the part of the MPinterfaces code designed to work with LAMMPS only prepared scripts to calculate the system energy, but later we added the functionality of accepting generic scripts, such as those to compute elastic constants. Using such a high-throughput framework we calculated the 21 distinct elastic constants^33^ using the script provided in the LAMMPS distribution version 15May2015. It is to be noted that the above script is one of the many other methods to calculate elastic constants and comparison and integration of other scripts with the one used here is subject to future work. Such a script allows to computation of elastic constant and energetics for any available empirical potential type and any crystal structure^34,35^. In our runs we used 10^−06^ as strain, 10^−10^ eV/Å for force convergence during the minimization to optimize the structure and 1000 maximum iteration for structure optimization. These are generalized computational set-up parameters, and the energetics and elastic constant data may or may not depend on them. We tested strain parameters for a range of values (10^−04^, 10^−06^ and 10^−08^) but obviously evaluating such set of parameters for all the calculations was too extensive a work and was not carried out here. The data for different strains (10^−04^, 10^−06^ and 10^−08^) for EAM potentials is given in the Supplementary Information as an example of testing of the script. For each structure and force-field, the code automatically generated the input structure file and input controlling parameters for energetics and elastic constant calculations. The relaxed structure was also stored along with the above files for later use such as for performing defect, phonon or other similar calculations. In some cases, (about 20 out of 3248) the jobs stopped running because of computer-cluster operation problems, so we manually reran them. Our current set up/scripts do not allow for checking of errors and resubmitting calculations automatically. Such an extension of the software is currently under work and will be part of a future project extension. Using the energetics information for each material, convex hull plot were drawn using the package provided in pymatgen^31^ (script named ‘PDPlotter.py’). All the input and output data for the calculations were saved in zipped and JSON format files for data accessibility and reproducibility. The JSON files are stored in a MongoDB database, which we found to be more advantageous over Structured Query Language (SQL) databases^36^. In MongoDB, the data is the schema and there is symmetry between the way data goes into the database and the way it comes out. A ‘ctcms_alloy.py’ script was used for all the ‘alloy’ class of force-fields. Similar ones were used for other force-fields. The scripts are provided on the github page.
3. **User-interface:** The data is presented in an interactive periodic table format at http://www.ctcms.nist.gov/~knc6/periodic.html. The four major aspects of our web interface are schematically shown in Fig. 1. First (panel a)) is the search box, where the user can type the element or element combination of interest. The interactive periodic table that appears as the web page is opened is shown in panel b). Here the user can input the query by simply clicking on the element or element combination of interest. In the example the binary alloy Ni-Al was chosen. Panel c) shows the header. At present it displays the calculation id (‘Calcs’), the Materials Project id for the structure (Mpid’), the formula for the system, the labels and units for the various computed quantities (energy per atom of the system, seven elastic constants, Voigt bulk and shear modulus, and energy above hull from DFT data (discussed later)). Lastly the name of the potential used is displayed (‘Forcefield’). Although only a few elastic constants are currently displayed in the table, all the elastic constants have been calculated and the ones not reported in the table can be obtained directly from the output files. The last panel (d) contains the actual data for all the quantities discussed in the header section. These data are interactive in nature. Clicking on the ‘Calcs’ tab downloads the zipped input and output files of the LAMMPS calculations. Clicking on the ‘MP-id’ tabs redirects to the Materials Project webpage for the same system, while clicking on ‘forcefield’ tab downloads the potential used in the calculation. Each zipped directory obtained by clicking on the ‘Calcs’ tab consists of following files: ‘data’ (the input structure coordinates and dimensions), ‘log.lammps’ (the file containing LAMMPS output), ‘restart.equil’ (the input file for restarting LAMMPS calculation if needed), ‘in.elastic’ (the input file for LAMMPS). The file ‘in.elastic’ is obtained by combining the three sub-files ‘init.mod’ (initializing calculation setup), ‘potential.mod’ (setting up of potential format and file), and ‘displace.mod’ (for taking into account all the displacements). To combine above-mentioned scripts ‘inelast.mod’ is used. These files were obtained from the LAMMPS source-code distribution. The ‘init.mod’ and the ‘potential.mod’ files were modified for specific structures and force-fields by the MPInterfaces code automatically while ‘displace.mod’ file was unchanged compared to the LAMMPS distribution. The only exceptions to this were the COMB and ReaxFF potentials, where specification of charge equilibration (‘qeq’) was needed. The relaxed structures are saved as ‘restart.equil’. To refresh the search, ‘Refresh’ key should be invoked.

All the data we computed is also available in JavaScript Object Notation (JSON)^37^ format, to facilitate a command interface for downloading the data instead of just a web-based interface.

**Figure 2:**
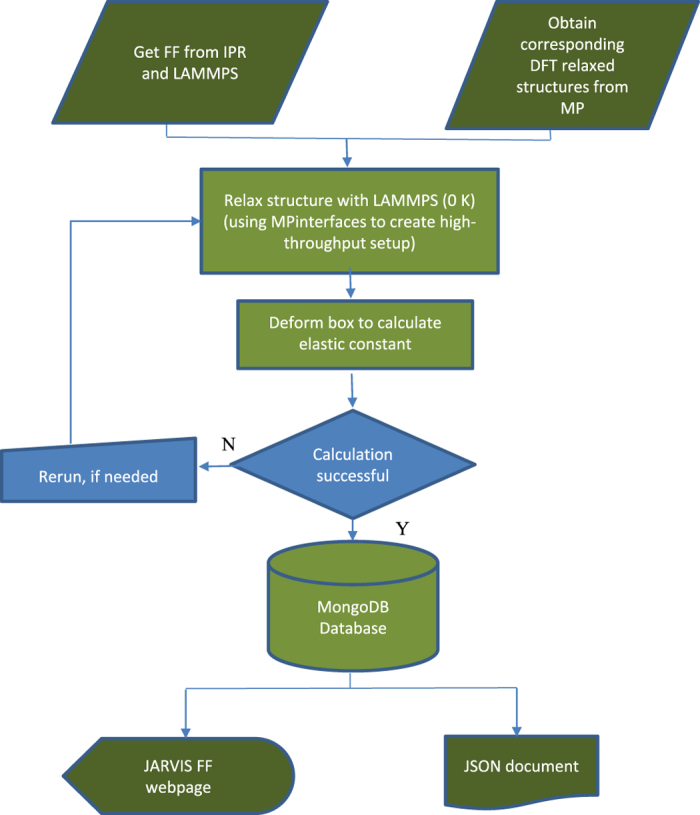
Flow chart used in the computation and storage of force-field data.

A flow-chart for the process we followed to compute and display all our data is given in Fig. 2.

### Code availability

The code is publicly available at the github link (https://github.com/JARVIS-Unifies/JARVIS-FF) and it is written in the python language. The code can be schematically divided in three major parts: 1) the assimilation of structures from the MP website^25^, and force-fields from the IPR website^3^, 2) the configuring of the high throughput calculation framework, and 3) the post-processing of the data. Step 2) heavily uses the MPinterfaces code^25^ to convert the DFT-format structure files into LAMMPS format, and to automatically prepare the input files for LAMMPS based on the specific structures at hand^38^. The Post-processing (step 3) is performed using normal file viewing or querying a JSON file. The work is under General Public License (GPL). The convex hull plot was obtained with the pymatgen distribution which uses composition and energy of the system. The script for calculating the convex hull using materials project is given at the github link. The reference DFT bulk modulus data was obtained from Materials Project elastic calculation data^33^. The data is available in JSON format at the github link. A back-up of the data is also kept at NIST’s materialsdata webpage https://materialsdata.nist.gov/dspace/xmlui/handle/11256/702. We compared the bulk modulus from LAMMPS to DFT wherever applicable. The script ‘get_entries_from_json.py’ can be used to plot the convex hull data. The input to the script is mainly the composition, e.g., Cu-H-O and the force-field, e.g., ‘ffield.CuOCH.comb3’.

## Data Records

All data computed in this work can be found at the github link https://github.com/JARVIS-Unifies/JARVIS-FF. The complete data set can also be downloaded in a JSON from Dryad Digital Repository (Data Citation 1). Key variables for the JSON file are shown in Table 1. They include energy per atom, elastic constants, associated structures and force-fields. We included energy/atom, instead of cohesive energy, to match the quantities provided in MP. For many potentials, however, the two energies coincide because of original potential fitting choices. Energy per atom values can be post-processed to calculate the cohesive energy and heat of formation of the system with respect to their corresponding reference state. The data is available in three forms: the JSON file, the html page (http://www.ctcms.nist.gov/~knc6/periodic.html) and the REST API services (available soon). The script for plotting the convex hull and fetching energetic/elastic constant information is provided on the github webpage mentioned above. The Pymatgen code is used for plotting the data. The convex hull plot uses the ‘composition’ and ‘totenergy’ parameter of the above JSON file for a particular system such as Ni-Al. The convex-hull^39^ plot hence obtained can be compared with data obtained from MP. More on this will be discussed in the Technical Evaluation section.

## Technical Validation

In this section, the reproducibility of the data in our database is discussed. We also demonstrate how to use these data to determine the range of applicability of the investigated force-field/potentials.

All the data in the database is intended to be reproduced very easily, as they were obtained by running the publicly available code LAMMPS and all the used input files are available for download from the zipped folders stored on the web for each calculation. Each folder contains the input controlling parameters, the structure file and the corresponding force-field. Combining the 3 files along with ‘displace.mod’ and ‘inelast.mod’ (provided on the github page and taken form LAMMPS source distribution) produces the input file LAMMPS needs to run the exact same calculation. These were obtained from LAMMPS software and are available at the github link https://github.com/JARVIS-Unifies/JARVIS-FF. A database of force-field evaluation with different version of LAMMPS as well as force-field can enable version control of the entire data.

To validate the FF applicability, the elastic constants and cohesive energy data of each structure are compared with those obtained using DFT, if available. These DFT data were taken from the Materials Project (MP) and were reported to be within ±15% of the experiments^33^. We found that given the computational set up parameters we used, many FFs produce elastic constants that differ significantly from their experimental values. This was not unexpected, as many FFs are not designed to reproduce mechanical deformations. Before giving detailed analysis of force-field data, a brief overview of FF types can be useful.

Embedded atom method (EAM) is one of most accurate predicting FFs for metals and alloys with relatively inexpensive calculations. In EAM potentials basic mechanical properties are always included in the fitting, making these potentials well suited for simulating mechanical deformations. As an example, Al EAM potential (Mishin-NiAl-2009)^40^ produces bulk modulus, C_11_, and cohesive energy of 78.9 GPa, 113.8 GPa and −3.36 eV/atom respectively. The bulk modulus, C_11_ and cohesive energy (E_c_) from DFT are 83.28 GPa, 103.93 GPa and −3.78 eV/atom, while the experimental values are 76 GPa, 113 GPa, and −3.360 eV/atom respectively. Hence, the classical mechanical properties data here remains within 5% of the experimental or DFT values. It is to be noted that DFT-single atom energy is typically strongly negative, but it is close to zero for most of the force-fields. Modified embedded atom method (MEAM) potential^41^ was developed to include directional bond characteristics to EAM potential, therefore allowing description of certain non-metallic systems. MEAM potentials such for SiC (P6_3_mc) has C_11_, Voigt-bulk modulus (B_v_) and cohesive energy (E_c_) as 211.1 GPa, 467.2 GPa and −6.43 eV/atom while the DFT values are 213.1 GPa,486.11 GPa, −7.5304 eV/atom respectively. Adaptive Intermolecular Reactive Empirical Bond Order (AIREBO)^18,19^ potential, which is generally fit for molecules, can capture certain mechanical properties, as in the case of graphite (P6_3_/mmc), where it gives C11, Voigt-bulk modulus (Bv) and cohesive energy (E_c_) of 899.1 GPa, 274.1 GPa and −7.47 eV/atom, to compare to the DFT values of 903.97 GPa, 236.46 GPa, and −9.2228 eV/atom respectively. Tersoff potentials^22,42,43^ are one of the most popular bond-order potentials for covalent systems. Tersoff potential for Si gives B_v_, C_11_ and cohesive energy (E_c_) of 97.8 GPa, 142.5 GPa and −4.63 eV/atom while DFT values are 83.01 GPa, 143.6 GPa and −5.4254 eV/atom respectively.

While all the above-mentioned force-fields do not contain charge information, ReaxFF force-field contains dynamic charge based on immediate environment. It is known to perform well for molecules as well as periodic structures. Fe $\text{Im}\bar{3}m$ in ReaxFF^44,45^ has B_v_, C_11_, as 173.1 GPa, 282.8 GPa and. while the DFT values are 182.46 GPa, 247.06 GPa respectively. The ReaxFF cohesive energy (E_c_) for Fe is −4.44 eV/atom, and the corresponding experimental value is −4.26 eV. In this case, MP only provides the energy per atom, not the cohesive energy, so a direct comparison is not possible. However other DFT evaluation of the iron cohesive energy validate the ReaxFF result, as in the case of the DFT work by Philipsen *et al*.^46^ where a value of −4.36 eV is reported for E_c_. Similar to ReaxFF, COMB potential is dynamic charge and bond-order based potential. It is known to work well for metallic, ionic and polymeric systems. Cuprous oxide in COMB^47,48^ has B_v_, C_11_ and cohesive energy (E_c_) as 102 GPa, 114.1 GPa and −3.65 eV/atom while the DFT values are 111.5 GPa,124.16 GPa and −4.79 eV/atom respectively. Again, it is to be noted these values can be sensitive to the initial calculation set-up and the target property/data for which the force-fields were fit. Now, we give some specific examples of FF data and the analysis of the force-field data as follows:

### 1) Single element: Al

Clicking on Al (or entering Al in search box) produces a list of results, one row for each of the 25 different force-fields available for Al. As the Material Project only has data for Al in face-centre cubic (FCC) structure (mp-134, space group $Fm\bar{3}m$), we also only list results for this one structure for different FFs. The energy per atom for all the potentials is around −3.36 eV/atom, with the exception of those of Al_zhou.eam^49^ (−3.58 eV/atom), Farkas_Nb-Ti-Al^50^ (−3.21 eV/atom), Al1.eam.fs (−3.41 eV/atom), Al90Sm10_Mendelev (−3.91 eV/atom), Al_wkg_MSME (−2.65 eV/atom). The names of the force fields are given here as they appear on the JARVIS-FF web-page, where the complete reference for each potential can be found. While it is beyond the scope of this work to analyse the reasons behind these outliers, we want to point out that energy differences are more important than the absolute energy/atom value, and, therefore, the variability of the above results may not have significant repercussions in the modelling of physical phenomena. However, the differences found in the elastic constant values among the various potential will have consequences when modelling mechanical deformations. Therefore, any user interested in modelling such phenomena should pay careful attention to the choice of force field. For instance, the C_12_ of Al is 61.6 GPa, while it is 123.6 GPa for Farkas_Nb-Ti-Al potentials using the above-mentioned calculation set-up parameters. With respect to bulk modulus values, all potentials for Al except for the Farkas-Al^50^ are within 2% of the experimental value (which is 76 GPa). This is to be noted that this potential was not mainly developed primarily for mechanical properties, so our web-interface can be used as an easy-look up table. Lastly, as there is only one structure for Al, the energy above hull is zero i.e., the structure in on the convex hull.

### 2) Binary system: Ni-Al

**Figure 3:**
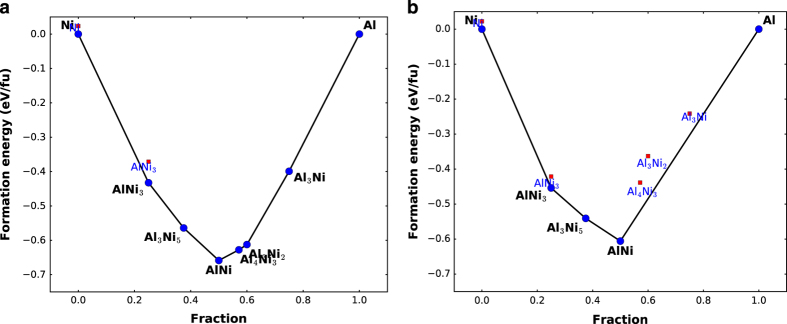
Convex hull plot for Ni-Al system using. (**a**) DFT (MP data), (**b**) Force-field (Mishin Ni-Al potential). Filled blue circles show stable while the red squares show unstable structures. Here ‘fu’ stands for formula unit.

As ‘Ni-Al’ is entered in search box or, alternatively, ‘Click Al +click Ni’ is selected, the webpage returns 49 rows of results because the Materials Projects has seven structures for the Ni-Al binary alloy and we found seven potentials for such compounds. Out of these seven, six compounds were stable or on convex hull (‘Energy_hull (DFT)’ (energy above hull from materials project’s DFT data) was 0.0 on webpage) The output is sorted based on formulae of structures to facilitate comparison of properties. As an example, results for Ni_3_Al, one of the Ni-Al primary phases (energy above hull 0.0), are found under the calculation id ‘Calc-178’. Clicking on this box downloads all the input and output files for calculation. Clicking on ‘mp-2593’ (the corresponding MP id) redirects to the MP data where the DFT energy/atom (−5.7024 eV) and elastic constant matrix can be found. This way the comparison between DFT and force-field data is effortless and immediate. In addition, the structure and Inorganic Crystal Structure Database (ICSD) data can be visualized in the materials-project webpage as well. The ICSD^51^ tag is an important link that can later be used to compare to other data resources such as ‘The Open Quantum Materials Database’ (OQMD)^52^ and ‘Automatic Flow for Materials Discovery’ (AFLOW)^53^, which is planned for future work. The ICSD tags are available at materials project webpages of the structures. It is to be noted that the DFT energy/atom was much lower than the potential data (−4.63 eV/atom), however the experimental value for this cohesive energy in −4.620 eV/atom^40^, that nicely validates the result. Also, these potentials are generally fit to experimental data. Similar searches can be made for ternary system as well. Next, convex-hull plot is used to know the phase stability of materials using their composition and energetics information. All the calculations done here have the information to generate a convex hull plot for DFT as well as force-fields. The data can be accessed using the script given in the github page. An example for Ni-Al system is given in Fig. 3. Here, the main low energy phases are similar for force-fields compared to DFT but high-energy structures (which are difficult to fit in force-fields) are not on convex-hull plot for force-fields. Again, this is due to the fact that the potential was not fitted for the energetics of the high energy phases. To reproduce the DFT convex hull plot, the potential would need to be parametrized. However, this was not the goal when the potential was made.

### 3) Ternary system (Fe-O-H, Cu-O-H):

**Figure 4:**
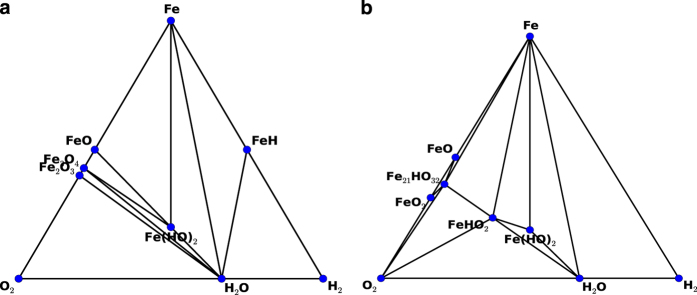
Convex hull plot for Fe-O-H system using. (**a**) DFT (MP data), (**b**) Force-field (Fe-O-C-H ReaxFF potential). Only stable structures are shown.

**Figure 5:**
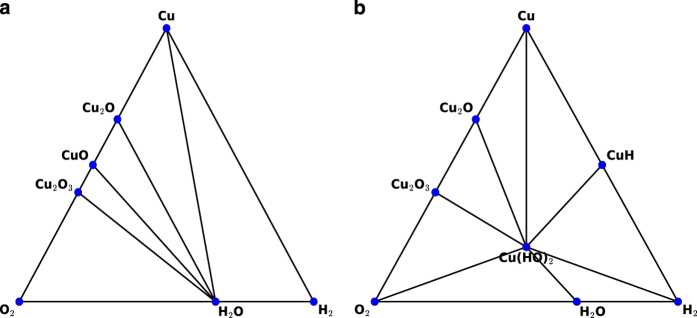
Convex hull plot for Cu-O-H system using. (**a**) DFT (MP data), (**b**) Force-field (Cu-O-H COMB potential). Only stable structures are shown.

Complex force-fields such as ReaxFF^44,45^ and COMB potentials^47,48^ are used for investigating heterogeneous systems. In these cases, it is generally common that the DFT convex hull plot and force-field convex hull plots are different. Examples for ReaxFF and COMB are shown in Figs 4 and 5. Here, only stable structures are shown for clarity. We notice that ReaxFF shows FeHO2, Fe_21_HO_32_, FeO_2_ are stable in force-field but not in DFT. Similarly, Cu (HO)2 and CuH are stable in COMB but not in DFT. This predicts that an MD simulation may predict different system behaviour than DFT-MD.

**Figure 6:**
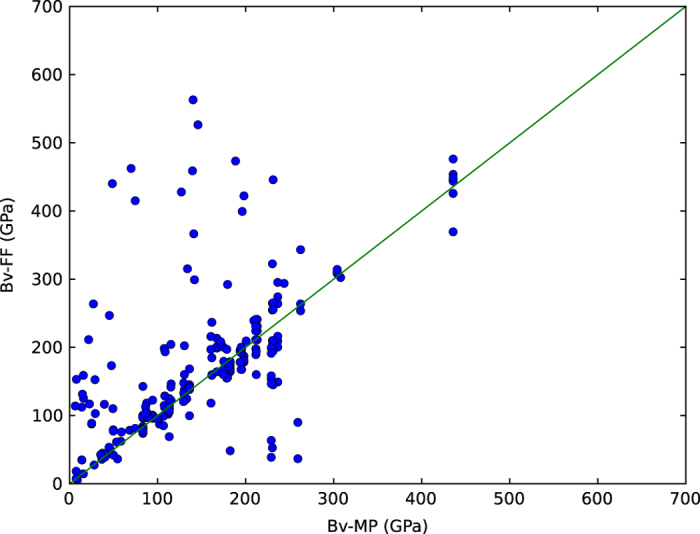
Comparison of Voigt-bulk modulus data of force-fields and DFT (materials project, MP data). The x-axis shows the bulk modulus for materials (for which FFs were available to us) from DFT calculation obtained through materials project, while the y-axis shows the bulk modulus for corresponding materials using the force-field calculations. The figure shows bulk moduli that correspond to structures that are common to JARVIS-FF and the Materials-project database. Because of this reason, out of 3248 entries in JARVIS-FF, only 408 entries are plotted.

Next, the FF and DFT Voigt-bulk modulus data were compared as shown in Fig. 6. The x-axis shows the bulk modulus for materials from DFT calculation obtained through materials project, while the y-axis shows the bulk modulus for corresponding materials using the force-field calculations. It is to be noted that potentials are generally fit to lattice constants, elastic constants of particular target materials and may not produce reasonable results for structures other than the ones they are fit to. For example, if a potential is fit to simulate Al-Cu binary system only, then it might not give very accurate description for elemental Al and Cu. In the Fig. 6 the slope of the curve is close to one for about 70% of the calculations (based on available DFT data) indicating many of the potentials were probably fitted to elastic constants.

### 4) Data analytics:

Data analytics tools provide the opportunity to determine correlations in data sets that can illuminate the accuracy of predictions and the flexibility of underlying models. To investigate if there are any trends in the elastic properties predicted by the various potentials, we perform a principal-component analysis on the relative errors of the predicted elastic constants compared to experimental data^7^ for the four FCC elements with the largest number of available potentials, i.e., Al, Cu, Ni, and Ag. In our analysis, we removed outliers with errors larger than 50% in *c*_11_ and *c*_12_ and larger than 100% in *c*_44_, as these indicate that the potentials were likely not optimized for the elastic properties.

**Figure 7:**
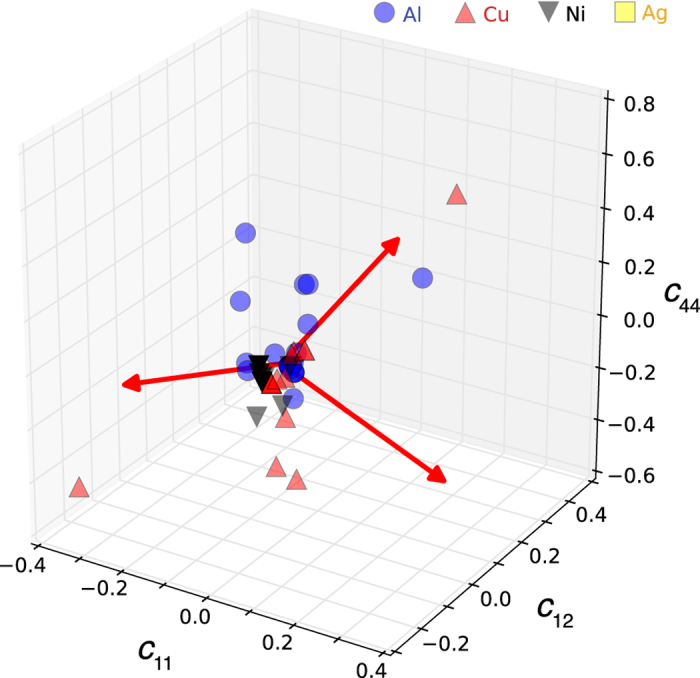
Relative errors of the elastic coefficients for the empirical potentials of the four FCC elements Al, Cu, Ni, and Ag compared to experimental values. The vectors illustrate the direction of the principal component of the data explaining the variance.

**Figure 8:**
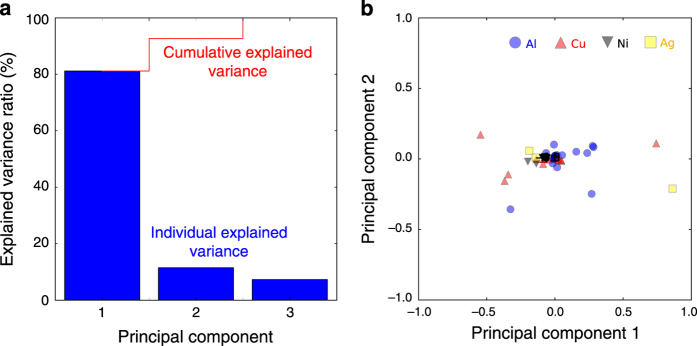
The results of the principal component analysis for the relative error of the elastic coefficients of the four FCC elements Al, Cu, Ni, and Ag show. (**a**) the explained variance for the three components and (**b**) the data transformed in the principal components and projected onto the first two components. The first eigenvector dominates the variance in the data.

Figure 7 shows the relative errors of the elastic coefficients of the four elements and illustrates the larger relative errors for *c*_44_, compared to *c*_11_ and *c*_12_. Figure 8a,b show the results of the principal component analysis, Fig. 8a shows the variance that is predicted by each of the three principal component eigenvectors and Fig. 8b the data projected onto the first two principal component eigenvectors. The results illustrate that most of the variance is explained by the first eigenvector (0.33, 0.31, 0.89) where the three components correspond to *c*_11_, *c*_12_, and *c*_44_. Thus, the principal component analysis for this subset of empirical potentials predicts a relative error in *c*_44_ of about 2.5 times the sum of the errors in *c*_11_ and *c*_12_. This example illustrates the usefulness of comprehensive datasets for predictions of fundamental materials parameters by empirical potentials and other models to aid in the selection and the development of empirical potentials.

The above results emphasize the need for judgement in the use of empirical potentials and motivates the design of better empirical potentials. Also, it is to be noted that the DFT databases have been recently made available, hence researchers have now access to data in order to compare their testing and development of potentials with the easy-accessible DFT results^54^.

## Usage Notes

The database presented here represents the to-date largest collection of consistently calculated properties of materials using interatomic potentials. We anticipate that this dataset, and the methods provided for accessing it, will provide a useful tool in fundamental and application-related studies of materials especially metallic and ionic systems for material design. Based on the list of potentials, user will be able to choose potential for their particular applications. Future applications will include the implementation of similar evaluation technique to polymer/bio-materials and on the fly calculation of properties on web by uploading or specifying particular structure. Data mining, data analytics, and machine learning tools then can be added to guide screening of materials.

## Additional Information

**How to cite this article:** Choudhary, K. *et al.* Evaluation and comparison of classical interatomic potentials through a user-friendly interactive web-interface. *Sci. Data* 4:160125 doi: 10.1038/sdata.2016.125 (2017).

**Publisher’s note:** Springer Nature remains neutral with regard to jurisdictional claims in published maps and institutional affiliations.

## Supplementary Material



Supplementary Information

## Figures and Tables

**Table 1:** JSON keys for metadata and their descriptions.

**Key**	**DataType**	**Description**
Search	string	for the system’s element type, e.g., Ni-Al
Mpid	string	materials project id, e.g., mp-134. Corresponding DFT data are available at https://www.materialsproject.org/materials/mpid
Totenergy	number	total energy of the system (unit eV)
Energy	number	energy per atom of the system (unit eV)
elastic_matrix	number	the elastic constant matrix (unit GPa)
Bv	number	Voigt bulk modulus (unit GPa)
Gv	number	Voigt shear modulus (unit GPa)
composition	string	the chemical composition of the system
Forcefield	string	the potential file used in the calculation

## References

[d1] Dryad Digital Repository Choudhary K. 2016 http://dx.doi.org/10.5061/dryad.dd56c

