# Supplementary material for: Evaluation and comparison of classical interatomic potentials through a user-friendly interactive web-interface
Source: Sci Data. 2017 Jan 31;4:160125. doi: 10.1038/sdata.2016.125 (PMC5283064; doi:10.1038/sdata.2016.125)
Supplement: Supplementary Information [file sdata2016125-s2.doc]

**Supplementary information: Evaluation and comparison of classical interatomic potentials through a user-friendly interactive web-interface**

Kamal Choudhary^1^, Faical Yannick P. Congo^1^, Tao Liang^4^, Chandler Becker^2^, Richard G. Hennig,^3^ Francesca Tavazza^1^

1. Materials Science and Engineering Division, National Institute of Standards and Technology, Gaithersburg, MD 20899, U.S.A
2. Office of Data and Informatics, National Institute of Standards and Technology, Gaithersburg, MD 20899, U.S.A
3. Department of Materials Science and Engineering, University of Florida, Gainesville, FL 32611, U.S.A.
4. Department of Materials Science and Engineering, The Pennsylvania State University, University Park, PA 16801, USA

The following figures show comparison of Voigt-bulk modulus data of force-fields and DFT (materials project, MP data) with different strains used from LAMMPS calculations.


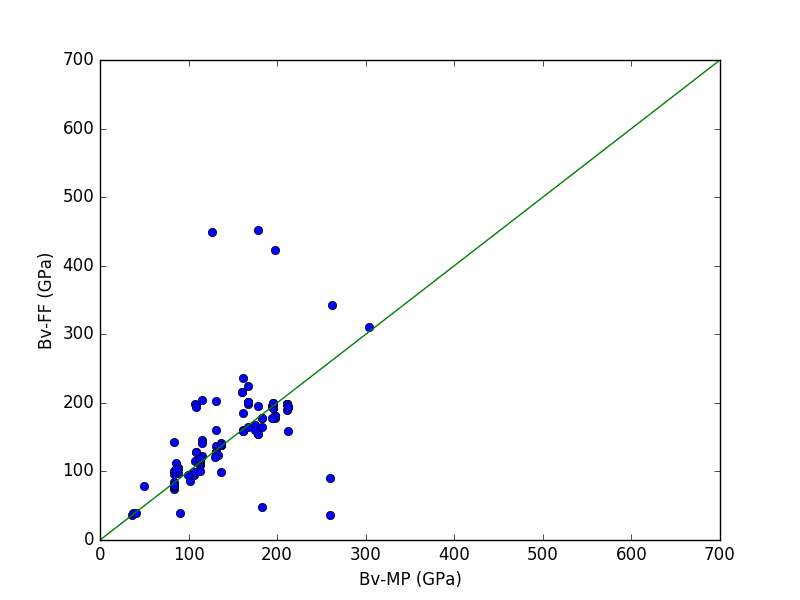


Fig. S1a The figure shows the bulk modulus data with 10^-4^ as strain used in LAMMPS calculations.


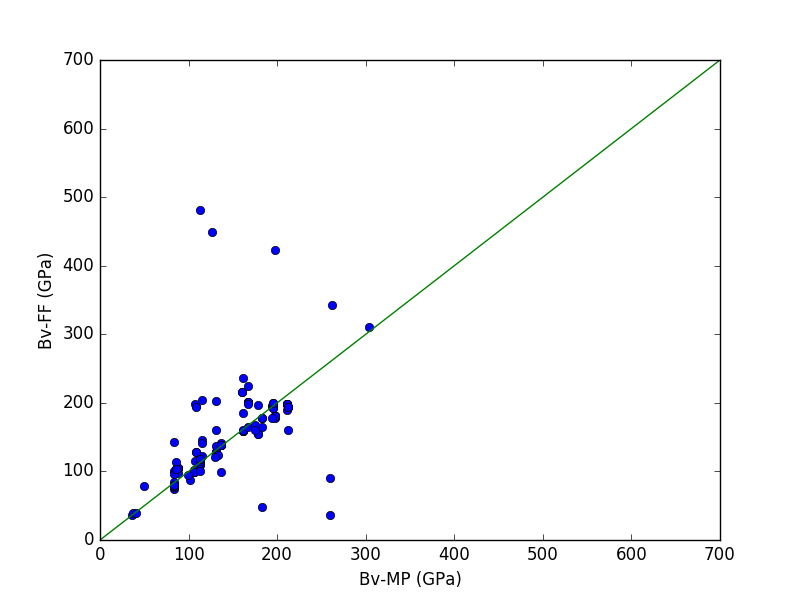
Fig. S1b The figure shows the bulk modulus data with 10^-6^ as strain used in LAMMPS calculations.


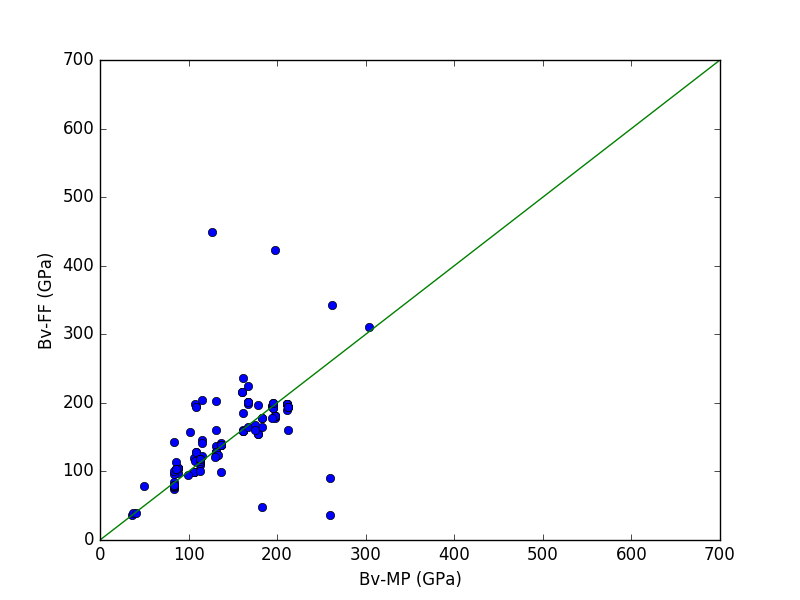


Fig. S1c The figure shows the bulk modulus data with 10^-8^ as strain used in LAMMPS calculations.
